# COVID-19 seroepidemiological survey among healthcare workers in the City of Ribeirão Preto, São Paulo, Brazil

**DOI:** 10.1590/0037-8682-0088-2022

**Published:** 2022-08-05

**Authors:** Patricia Martinez Évora, André Machado Siqueira, Rodrigo Guerino Stabeli

**Affiliations:** 1 Fundação Oswaldo Cruz, Plataforma Bi-institucional de Medicina Translacional, Ribeirão Preto, SP, Brasil.; 2 Fundação Oswaldo Cruz, Instituto Nacional de Infectologia Evandro Chagas, Rio de Janeiro, RJ, Brasil.

**Keywords:** COVID-19, Healthcare workers, Seroepidemiological survey, Ribeirão Preto, Brazil

## Abstract

**Background:**

Coronavirus disease (COVID-19) serology testing evaluates the prevalence of COVID-19 cases.

**Methods:**

A seroepidemiological survey of COVID-19 among healthcare workers was performed (June 2020 to November 2020) in Ribeirão Preto, São Paulo, Brazil. Overall, 10,172 and 2,129 workers participated in the first and second phases, respectively.

**Results:**

First phase: 12.7% tested positive for COVID-19 (73.5% females and 35.2% aged 30-39 years), and 29.6% were nursing technicians. Second phase: 12.1% tested positive for COVID-19 (65.5% females and 33.3% aged 40-49 years), and 24.8% were nursing assistants.

**Conclusions:**

In 2020, healthcare workers in Ribeirão Preto had COVID-19 in a similar way.

The new coronavirus disease (COVID-19) was reported in December 2019 in an outbreak of pneumonia of unknown cause associated with a visit to a seafood market in Wuhan, China^1^. From China, the virus quickly spread to other countries worldwide and on January 30, 2020, the World Health Organization (WHO) declared that the ongoing epidemic constituted a Public Health Emergency of International Importance (ESPII)^2^. Despite the declaration of ESPII and the measures adopted to contain viral transmission, the number of cases and affected countries continued to increase, and on March 11, 2020, when the number of confirmed cases had reached 128,400, the WHO declared the COVID-19 a pandemic.

Healthcare workers are at risk of COVID-19. A meta-analysis of 11 studies revealed that the proportion of healthcare workers who were positive for COVID-19 among all COVID-19 patients was 10.1%, but the severity and mortality among them were lower than those in the other patients^3^. Therefore, the prevention of infections among healthcare workers is important to reduce not only morbidity and potential mortality but also secondary transmission^4^. 

Epidemiological surveillance of COVID-19 cases captures only a proportion of all infections, as the clinical manifestations of the infection are highly variable, ranging from severe and life-threatening to being asymptomatic. Thus, considering that a seroepidemiological study provides information on the proportion of the exposed population, the present project, which is part of a multicenter study of the natural history of the new coronavirus in Brazil (REBRACOVID), conducted from June 2020 to November 2020 a seroepidemiological survey among healthcare workers in Ribeirão Preto, São Paulo, Brazil, with the aim of helping the health surveillance system to respond in a timely manner to the challenges faced during the pandemic. Nowadays, the survey may also assist in the comparison between COVID-19 variants from June 2020 to the present day.

The present study aimed to (i) estimate the number of healthcare workers who have had close contact with COVID-19 patients; (ii) evaluate the distribution of cases by sex, age group, and profession/occupation among healthcare workers; (iii) estimate the number of infected healthcare workers with chronic disease; (iv) estimate the number of asymptomatic infected healthcare workers; (v) describe the clinical manifestations of COVID-19 in healthcare workers.

Healthcare professionals (i.e., physicians, nurses, nursing technicians, nursing assistants, physiotherapists, and dentists) as well as support staff (i.e., receptionists, cleaning assistants, security, and administrative assistants) who work in basic care units, emergency care units, psychosocial care centers, family health units, and hospitals could participate in the study, regardless of the presence of flu-like symptoms or level of exposure to the virus. The main inclusion criterion was working for at least 4 consecutive hours in healthcare units or hospitalization of patients with confirmed COVID-19. The adherence to the study was spontaneous, and approximately one-third of the total number of individuals working in the health area in Ribeirão Preto participated in the first phase. Employees from the Municipal Health Department of Ribeirão Preto were on duty at the city's health units where COVID-19 patients were being treated, and healthcare workers who were interested in testing for COVID-19 approached them, signed the consent form, and filled out the Health Surveillance Department survey. A blood sample was then collected from the participants, properly stored, and taken to the municipal laboratory where an IgG serological test using the Abbott ARCHITECT severe acute respiratory syndrome coronavirus 2 (SARS-CoV-2) IgG assay was performed. The SARS-CoV-2 IgG assay, in turn, is a chemiluminescent microparticle immunoassay used for the qualitative detection of IgG antibodies to SARS-CoV-2 in human serum and plasma.

In the first phase of the seroepidemiological survey, data from the surveys of 10,172 participants who underwent the IgG serological test for COVID-19 between June 29 and August 26, 2020, were analyzed. In the second phase, the surveys completed by 2,129 participants between October 2 and November 3, 2020, were analyzed. Access to test results and surveys was kindly provided by the city hall of Ribeirão Preto.

Among the 10,172 healthcare workers who performed the IgG serological test for COVID-19 in the first phase of the study, 1,291 tested positive, the equivalent to 12.7% (Table 1). In the second phase, a total of 2,129 healthcare workers participated, of which 1,942 (91.2%) had also participated in the first phase. Moreover, of the total number of participants in the second phase, 258 (12.1%) tested positive for COVID-19 (Table 1). 

**TABLE 1:** Distribution of sex, age, and chronic disease presence in healthcare workers of Ribeirão Preto, São Paulo, Brazil, who tested positive for COVID-19 in the first and second phases of the study. Ribeirão Preto/SP, Brazil, 2020.

	First phase	Second phase
	(n=1,291)	(n=258)
	(+) COVID-19	(+) COVID-19
**Female**	949 (73.5%)	169 (65.5%)
**Male**	342 (26.5%)	89 (34.5%)
**18-19 years old**	5 (0.4%)	0 (0.0%)
**20-29 years old**	275 (21.3%)	20 (7.7%)
**30-39 years old**	455 (35.2%)	63 (24.4%)
**40-49 years old**	347 (26.9%)	86 (33.3%)
**50-59 years old**	167 (12.9%)	66 (25.6%)
**60+ years old**	42 (3.2%)	23 (8.9%)
**Comorbidity**	1 comorbidity: 185 (14.3%)	1 comorbidity: 57 (22.1%)
	+1 comorbidity: 55 (4.3%)	+1 comorbidity: 22 (8.5%)
**Systemic arterial hypertension**	123 (41.1%)	46 (44.2%)
**Diabetes/pre-diabetes**	43 (14.4%)	18 (17.3%)

**COVID-19:** coronavirus disease.

Considering gender and age group, in the first phase, 949 (73.5%) participants who tested positive for COVID-19 were women and 455 (35.2%) were individuals aged 30-39 years (Table 1). In the second phase 169 (65.5%) of the participants who tested positive for COVID-19 were women and 86 (33.3%) were individuals aged 40 - 49 years (Table 1).

As for presence of chronic disease, 240 (18.6%) participants who tested positive for COVID-19 in the first phase reported having chronic disease, with 55 (4.3%) having more than one comorbidity (Table 1). In the second phase, 79 (30.6%) participants who tested positive for COVID-19 reported having chronic disease, with 22 (8.5%) having more than one comorbidity (Table 1). Considering the chronic diseases, systemic arterial hypertension was present in both phases, affecting 123 (41.1%) and 46 (44.2%) of the participants who tested positive for COVID-19 in the first and second phases, respectively (Table 1). Diabetes/pre-diabetes was the second most prevalent comorbidity, affecting 43 (14.4%) of the individuals who tested positive for COVID-19 in the first phase and 18 (17.3%) of the participants in the second phase (Table 1).

Among professions, nursing technicians were accounted for 382 (29.6%) individuals who tested positive for COVID-19 in the first phase, whereas 143 (11.1%) were nursing assistants, 127 (9.8%) were physicians, and 122 (9.4%) were nurses. In the second phase, 64 (24.8%) individuals who tested positive for COVID-19 were nursing assistants, whereas nurses and physicians accounted for 24 (9.3%) participants each (Figure 1).

**FIGURE 1:**
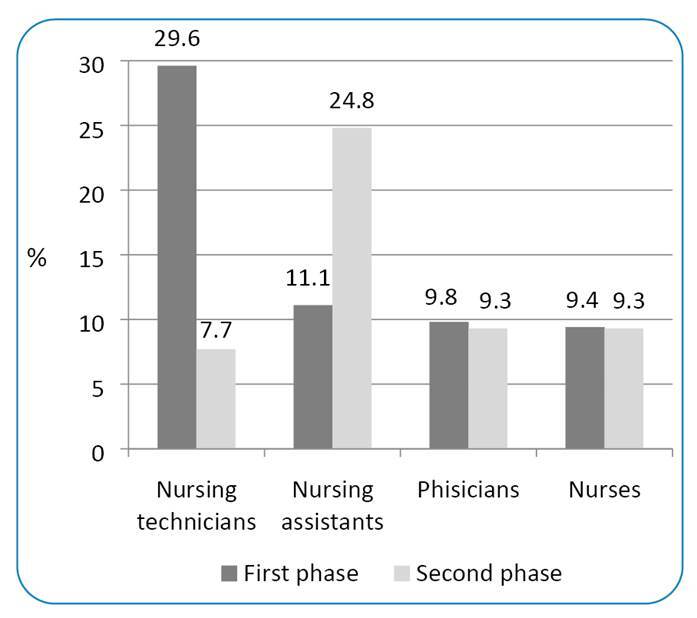
Distribution of the number of healthcare workers who tested positive for coronavirus disease (COVID-19) in Ribeirão Preto, São Paulo, Brazil, in the first and second phases of the study, by profession.

Of the 1,291 study participants who tested positive for COVID-19 in the first phase, 409 were asymptomatic, the equivalent to 31.7% (Table 2). Moreover, in the first phase, among 882 (68.3%) individuals who presented one or more symptoms of COVID-19, the most common symptom was headache (675 participants; 52.3%), followed by body pain (603 participants; 46.7%) and ageusia (loss of taste; 566 participants; 43.8%) (Table 2). Of the 258 participants who tested positive for COVID-19 in the second phase, 95 were asymptomatic, the equivalent to 36.8% (Table 2). Among the 163 (63.2%) individuals who presented one or more symptoms of COVID-19, the most common symptom was headache (118 participants; 45.7%) participants, followed anosmia (loss of smell; 101 participants; 39.1%) and body pain (98; 38%). 

**TABLE 2:** Manifestation of COVID-19 symptoms among healthcare workers from Ribeirão Preto, São Paulo, Brazil, who tested positive for the disease in the first and second phases of the study. Ribeirão Preto/SP, Brazil, 2020.

	First phase	Second phase
	(n=1,291)	(n=258)
	(+) COVID-19	(+) COVID-19
**Asymptomatic**	409 (31.7%)	95 (36.8%)
**Symptomatic**	882 (68.3%)	163 (63.2%)
**Headache**	675 (52.3%)	118 (45.7%)
**Body pain**	603 (46.7%)	98 (38%)
**Ageusia**	566 (43.8%)	94 (36.4%)
**Anosmia**	552 (42.8%)	101 (39.1%)

**COVID-19:** coronavirus disease.

A seroepidemiological survey aims to estimate the presence of antibodies in certain populations to understand how many people have already been exposed to a pathogen and how many can still become infected. The two phases of the present study had similar percentages regarding the number of people who tested positive for COVID-19 in the serological test offered by the Municipal Health Department of Ribeirão Preto. The first and second phases of the study demonstrated that 12.7% and 12.1% of participants, respectively, tested positive for COVID-19. With a similar result, a study conducted in Massachusetts, USA, found that 14% of healthcare workers tested positive for COVID-19^5^. In another study conducted among healthcare workers in Iran, only 5.6% tested positive for COVID-19^6^.

Regarding gender, both phases had a higher percentage of women. While the first phase revealed that 73.5% of women tested positive for COVID-19, the second phase reported 65.5%. These results are possibly because most of the healthcare professionals on the frontline, such as nurses, nursing technicians, and nursing assistants, are women. In agreement, a review and meta-analysis performed by Gholami et al.^7^ in healthcare workers showed a higher percentage of women with COVID-19 (78.6%). Another study conducted in a university hospital in São Paulo showed that 71.8% of women had COVID-19^8^.

Regarding age, there was a difference between the two phases. While the first phase had a higher percentage of individuals aged 30-39 years who tested positive for COVID-19 (35.2%), the second phase mainly comprised individuals aged 40-49 years (33.3%). This difference in results is possibly because the first phase had greater participation of professionals than the second phase. Gholami et al.^7^ and Faíco-Filho et al.^8^ reported that the average age of workers with COVID-19 was 38.7 years and 39.2 years, respectively. Furthermore, a study conducted in Iran showed that 84.5% of healthcare workers who tested positive for COVID-19 were aged 25-45 years^6^. In the first phase of the study, 18.6% of participants who tested positive for COVID-19 claimed to have chronic disease, with systemic arterial hypertension being the most common (41.1%), followed by diabetes/pre-diabetes (14.4%). The second phase of the study had 30.6% of participants positive for COVID-19 who had some comorbidity. Similar to the first phase, the most commonly reported diseases were systemic arterial hypertension (44.2%) and diabetes/pre-diabetes (17.3%). A study performed in King County, USA, showed that 47.9% of healthcare workers had some chronic disease, but the diseases were not specified^9^. In a review and meta-analysis, Gholami et al.^7^ showed that 18.4% of professionals had comorbidity, with hypertension being the most common (2.5%), followed by heart disease (2.5%) and chronic obstructive pulmonary disease (2.4%). 

As for profession, there was a difference between the first and second phases of the seroepidemiological survey. While the first phase showed that nursing technicians were the most affected by COVID-19 (29.6%), the second phase suggested that nursing assistants were the most affected (24.8%). This difference is possibly because only the first phase included the city's hospitals, where the highest concentration of nursing technicians is found. The percentage of physicians was similar in the first and second phases (9.8% and 9.3%, respectively), as was the percentage of nurses (9.4% and 9.3%, respectively). In accordance with the first phase (which included hospitals in Ribeirão Preto), Faíco-Filho et al.,^8^ who conducted the study in a university hospital in the city of São Paulo, showed greater involvement of nursing technicians toward COVID-19 (44%), followed by nurses (29%) and physicians (24.2%). Finally, Sabetian et al.^6^ observed that most cases of infection occurred among nurses (51.3%), which suggests that, worldwide, the most infected professionals are those on the frontline.

From the present seroepidemiological survey, it was observed that throughout 2020, COVID-19 continued to infect healthcare workers in Ribeirão Preto in a similar way. Furthermore, a considerable percentage of these professionals were asymptomatic, which makes the investigation of infection among these workers essential to reduce the spread of the virus. In agreement, a study conducted among healthcare workers in Iran showed that 35.5% of the participants were asymptomatic^6^. Another study in China showed that 78% of people infected were asymptomatic^10^. These data are important because asymptomatic carriers can transmit the disease and should therefore be considered a source of infection. Moreover, nowadays, the survey may also assist in comparing COVID-19 variants from June 2020 with those in the present day. Finally, considering that COVID-19 continues actively, protective measures for healthcare professionals remain essential both for self-protection and for decreasing infection rates among colleagues and family members. 
